# Human Stem Cell-Based Embryo Models in Implantation Research: Regulation, Consistency and Potential

**DOI:** 10.3390/biom15091287

**Published:** 2025-09-05

**Authors:** Søren Holm

**Affiliations:** 1 Centre for Social Ethics and Policy, School of Law, University of Manchester, Manchester M13 9PL, UK; soren.holm@manchester.ac.uk or soren.holm@medisin.uio.no; 2 Center for Medical Ethics, Institute of Health and Society (HELSAM), University of Oslo, 0318 Oslo, Norway

**Keywords:** embryo, embryo research, potential, regulation, stem cell-based embryo model

## Abstract

The use of human stem cell-based embryo models (hSCBEM) in implantation research is developing rapidly. This raises regulatory and ethical issues as these models become more complex and get closer to morphological and functional identity with human embryos. This paper provides an analysis of two possible approaches to resolving the regulatory issues. The first approach is to try to achieve consistency with current regulation of embryo research, and the second approach is to elaborate the regulation of hSCBEMs based on their developmental potential. It is shown that both approaches are problematic. The consistency approach is problematic because the current regulation of embryo research is best understood as being the result of a historical, political compromise in most jurisdictions. And the approach based on assessment of developmental potential is problematic because of unavoidable epistemic uncertainty about the potential of a new hSCBEM, and because of problems in determining what constitutes a particular model, and what changes to a model makes it into a different model.

## 1. Introduction

The field of human stem cell-based embryo models (hSCBEMs) is developing rapidly. Many different models are being developed, and they are used for researching a wide variety of research questions in relation to early embryo development. Using the label of hSCBEM for the whole class of models being developed is potentially misleading because many of the models are not models of ‘the embryo’ but models of a particular aspect of embryonic development. This means that there are many hSCBEMs that raise no specific ethical issues related to their intrinsic moral status, even for those who view the embryo as ethically valuable and who will be worried by the development of more complete embryo models.

There are, however, hSCBEMs also being developed with the explicit aim of developing a model that faithfully reproduces all aspects of embryo development, with a final goal of having a model that is indistinguishable from an embryo created by fertilisation [1,2,3,4,5,6]. If such an hSCBEM can be developed it will raise obvious ethical concerns because although it will have a different origin than an embryo created by fertilisation, it will be structurally and functionally identical [7,8].

One particular line of research uses hSCBEMs to study the attachment and implantation processes that are necessary for the establishment of a sustainable pregnancy in humans [3,9,10]. Such research will increase our understanding of the causes of implantation failure which is a common problem in humans. The research may eventually lead to new prevention or treatment modalities for implantation failure, or to new methods of contraception.

From an ethical and regulatory perspective, hSCBEMs used to study attachment and implantation are of particular interest since they will, in most cases, contain both embryonic and extraembryonic structures. The International Society for Stem Cell Research (ISSCR) made a distinction between integrated and non-integrated stem cell-based models in its 2021 guidelines and stated that the creation and use of integrated stem cell-based models should receive a higher level of ethical scrutiny. It defined integrated stem cell-based models as follows:

“These stem cell-based embryo models contain the relevant embryonic and extraembryonic structures and could potentially achieve the complexity where they might realistically manifest the ability to undergo further integrated development if cultured for additional time in vitro. Integrated stem cell-based embryo models could be generated from a single source of cells, for example expanded potential human pluripotent stem cells capable of coordinately differentiating into embryonic and extraembryonic structures. Alternatively, integrated stem cell-based embryo models could also be generated through the formation of cellular aggregates where extraembryonic/embryonic cells from one source are combined with embryonic/extraembryonic cells from different sources to achieve integrated human development. This might include using non-human primate cells as one of the sources.”

[11] (p. 2).

Many of the hSCBEMs being developed and used in implantation research will fall within this category, especially those used for studying the later stages of implantation where the ability of the extraembryonic tissues to sustain normal development of the embryonic structures become an important topic of study.

How should hSCBEMs in implantation research be regulated? There are two main ways in which this question can be answered. It can be answered by taking current regulation of embryo research as a starting point and aiming at regulatory consistency between current embryo research regulation and future hSCBEM regulation. It can also be answered by taking ethical considerations as the starting point and asking how they apply to various types of hSCBEM. This paper will explore both approaches and argue that they are both problematic in the sense that they are unable to provide definitive answers to the regulatory questions. The exploration will use the methods of conceptual and philosophical analysis.

It will first consider the problems in seeking to resolve the question as a question of regulatory consistency and then look at whether it can be resolved by one particular line of ethical argument that is implicit in the ISSCR guidelines and also plays a significant role in the guidelines from the Europeans Society for Human Reproduction and Embryology (ESHRE) [12,13], i.e., the so-called ‘argument from potential’.

## 2. Regulatory Consistency

The use of embryos for research is regulated in many countries, and this raises questions of regulatory consistency between the regulation of embryo research and the regulation of hSCBEM research. The exact details of the consistency questions that are raised will be jurisdictionally specific. Jurisdictions, for instance, differ in how they define the embryo in legislation and therefore differ in whether or not all or some hSCBEMs are already captured by that definition. It is not clear whether the circular definition in Section 1 (1) of the UK Human Fertilisation and Embryology Act 2008 of an embryo as “a live human embryo” captures any hSCBEMs [14], but the Australian and Dutch legal definitions plausibly do. Australian legislation defines the embryo as follows:

““*human embryo*” means a discrete entity that has arisen from either: (a) the first mitotic division when fertilisation of a human oocyte by a human sperm is complete; or (b) any other process that initiates organised development of a biological entity with a human nuclear genome or altered human nuclear genome that has the potential to develop up to, or beyond, the stage at which the primitive streak appears; and has not yet reached 8 weeks of development since the first mitotic division”

[15] (Section 7).

The Netherlands Embryo Act 2002 defines it as

“a cell or connected system of cells that has the capacity to develop into a human being”

[16] (p. 5).

It is important for the later exploration of the argument from potential to note that these legal definitions explicitly refer to either ‘potential’ (part b of the Australian definition) or ‘capacity to develop’ (the Dutch definition).

Some jurisdictions only allow research on surplus embryos from IVF (e.g., Japan), whereas others allow the creation of embryos for research (e.g., the UK). [17] Nearly all jurisdictions that regulate embryo research only allow embryos to be used in research for specific purposes, but the lists and definitions of those purposes differ between jurisdictions. Similar consistency questions may arise in relation to any funding restrictions that apply to embryo research, such as in relation to US federal funding or funding from the EU.

How should we think about and potentially resolve these regulatory consistency questions? A natural starting point would be to ask why we regulate human embryo research differently than other types of research, e.g., other research using tissue cultures. This question can be answered in two distinct, but interconnected ways. The first answer would focus on the historical context in which embryo research came to be regulated. Prior to the first successful IVF pregnancy in 1978, embryo research took place but was not regulated, primarily because it went under the regulatory radar. However, the success of IVF as a treatment for infertility and the immense media attention the birth of Louise Brown received made it evident that IVF itself had to be regulated, and that the underlying research using embryos also had to be regulated, since continuing research was necessary for the further development and optimisation of IVF techniques. In this context, IVF was conceptualised as a medical treatment for a reproductive problem and embryo research as a necessary enabler of this treatment [18]. Given this framing, the regulatory question therefore became not whether embryo research should be allowed, but what kinds of embryo research and under what conditions. The second answer to the regulatory question would focus on what makes the human embryo a particular object of regulatory concern and protection. This question had been discussed in relation to the foetus in the abortion debates in the 1960s and early 1970s and a number of lines of ethico-legal arguments had been developed leading to widely diverging conclusions. These lines of argument were essentially repurposed for the new discussion of embryo research. At one end of the spectrum, ‘personhood’ theorists argued that the foetus and a fortiori the embryo has no intrinsic moral status or importance because it does not have any of the features that makes a person morally valuable, e.g., self-consciousness, or a conscious desire to keep on living [19,20,21,22,23,24,25]. On this view, there is nothing ethically problematic about embryo research for any bona fide scientific purpose, and nothing problematic in creating embryos specifically for research. At the other end of the spectrum, the main philosophical line of argument was based on what became known as the ‘argument from potential’ [26]. This argument, which I will explicate further below, proceeds from the idea that what gives a foetus and an embryo moral status is that it has the intrinsic, active potential to develop into a born human being. It is, in the ethically relevant sense, already one of us with a future like ours [27]. It is rather obvious that most jurisdictions eventually regulated embryo research in ways that are incompatible with both of the positions outlined just above. Types of embryo research were allowed in many jurisdictions which are incompatible with the argument from potential (e.g., creating embryos for research), but research was restricted in ways that are incompatible with the personhood line of argument. Some have tried to explain this as based on a concept of ‘respect’ for the human embryo. This view is, for instance mentioned in the 1984 Warnock report: “We found that the more generally held position, however, is that though the human embryo is entitled to some added measure of respect beyond that accorded to other animal subjects, that respect cannot be absolute, and may be weighed against the benefits arising from research” [28] (p. 62). But unless ‘respect’ can be explicated quite precisely, this seems unsatisfactory as an explanation of the details of any particular set of regulations [29,30,31]. For instance, how is allowing the embryo to be destroyed in research showing respect? Or, if research is allowed to take place, is any kind of research, apart from the obviously trivial or otherwise unethical research, ‘disrespectful’ in any way? It is therefore probably better to see the initial regulation of embryo research as a historical and localised political compromise between an IVF-driven research imperative on the one hand and, to some extent, an inchoate view that human embryos have moral value and should be protected on the other hand (for instance see the accounts of the UK developments [18,32]). It is, at best, a political result of the type that Sunstein describes as an incompletely theorised agreement where all parties to the agreement can live with and rationalise the result within their own particular justificatory framework, but where there is still fundamental disagreement about which underlying justification is the right one [33]. This is not unusual in relation to regulations of ethically contentious issues in pluralistic societies and can also be seen in relation to, for example, abortion legislation. Ethics is far from the only relevant consideration when deciding on the content and design of regulations that can be successfully implemented in highly complex social and political contexts [34,35].

However, our inability to identify a specific theoretical justification for our current regulation of embryo research in most jurisdictions that permit some kind of research leads to a situation where it is difficult to make principled judgments about new types of proposed research or, as in the case of hSCBEM, new objects for regulatory attention. Usually, we would apply some type of consistency argument, based on a series of analogies between the new regulatory issue and our current regulations, but in relation to embryo research, we lack a stable basis for such analogies. The depth of that problem is illustrated in the recent argument by Emma Cave that even hSCBEMs that are known to be morphologically and functionally identical to human embryos, i.e., perfect embryo replicates should not be regulated as human embryos [36]. This amounts to the claim that two entities that are qualitatively indistinguishable from each other should not be treated in the same way for the purposes of regulation and seems clearly incompatible with any attempt to reach regulatory consistency.

There are also more practical problems in applying consistency arguments to hSCBEMs, since embryos and hSCBEMs are created in different ways, and do not necessarily develop at the same speed. The so-called 14-day rule, which is a feature of the regulation of embryo research in many countries, is, for instance, not directly applicable to hSCEBMs.

In relation to the regulation of contentious questions, the views of the public or of other stakeholder groups may be important in order to strike the right balance between countervailing considerations. However, the views of the public or other stakeholders cannot be determinative as to whether a particular policy option should be implemented. It is a trivial observation that the majority of the public may simply be wrong in certain cases, and that the views of other stakeholders may be influenced by the particular nature of the stake they hold. It is thus important to know what the public and other stakeholders think about using hSCBEMs in implantation research when properly informed, and it is important to know how these views differ from or correlate with their views on embryo research, but these views can only be one of the inputs into the policy-making process. How the public views a particular entity, e.g., in relation to what level of respect it should be afforded, may be unrelated to the ethico-scientific reasons for treating the entity in a certain way in regulation. The public may have different views on the respect that should be afforded to guinea pigs and to rats, but it would be highly problematic to use that as a reason to regulate research using these two different species of rodents differently.

## 3. The Argument from Potential

As noted in the previous section, it is by no means certain that an analysis of the relevant similarities and dissimilarities between human embryos and hSCBEMs will resolve the regulatory consistency questions. Our current regulation may not be based on any particular, identifiable set of ethically or legally justified distinctions, arguments and conclusions, but may be a compromise emerging from a historically specific political process. This is, as already mentioned, not necessarily a criticism of the actual regulation since a lack of direct translation from ethics to regulation is ubiquitous in the regulation of ethically contentious areas and also to be expected, given that regulation has to be implemented in highly complex social systems, and is subject to path dependency [35].

In the following section, I will explicate the argument from potential and its possible implications for the moral status of hSCBEM, and the downstream regulatory implications. I am focusing on this line of ethical argument because it is probably the one that best explains why embryo research should be regulated and restrictions put on such research. There is a very large body of literature on the argument from potential in bioethics, and on the concept of potential in general, and it is impossible to cover all of the arguments and counterarguments in the following. A good introduction to the intricacies of the debate in bioethics is the 2014 book edited by Lizza [37], and the 2018 Springer ‘Handbook of Potentiality’ gives an overview of the broader philosophical discourse on potentiality [38].

The argument from potential has two parts: the first part defining the potential of an entity, and the second part linking that potential to ethical evaluation.

What do we mean when we say that an embryo or an hSCBEM has the potential to become a born human being? Or more generally, what do we mean when we say that X has the potential to become Y? There are two main ways of understanding potential. The first and most commonly defended in the philosophical literature is to analyse statements about potential in terms of dispositions [39,40]. On this analysis, a glass vase is fragile, i.e., has the potential to break, if it has a disposition to break in relevant circumstances. The second is to analyse potential as a modal operator in modal logic alongside operators such as ‘necessary’ and ‘possible’, modal logic being the branch of formal logic that investigates the truth conditions for statements containing claims about necessity and possibility [41,42]. On both accounts, potential can be intrinsic, i.e., based solely on the properties of the entity in question, or it can be extrinsic, or it can be both intrinsic and extrinsic. And both accounts make a distinction between potential and possibility. It is possible to break a sturdy glass vase if sufficient force is applied, but that does not show that the sturdy vase had a disposition to break or that it was intrinsically fragile. Finally, a potential can be active as in the case of some potentials of living entities or it can be passive as in the case of the fragile glass vase. So, in order for an entity to have an active, intrinsic potential to become something else, it is not enough that it is merely possible for that change to happen, it must happen at least partly due to the intrinsic, active causal powers of the entity in question. On the other hand, that the change is unlikely to happen does not in itself show that the entity did not have the potential or disposition. A well-packaged, thin champagne flute is still intrinsically fragile, even if it is unlikely to break if it stays in the packaging. Its disposition to break is merely temporarily masked. Or to take another example, most acorns never become oak trees, but they all at some point have the potential to become one.

On this account of potential, the human embryo has an active, intrinsic potential to become a born human being. We know this to be the case because many human embryos do become born human beings.

The second part of the argument aims at establishing a justification for linking potential and ethical valuation. It proceeds from the assumption that it is, or ought to be, uncontroversial that there is a class of entities that have full moral status including the right not to be killed and that all adult human beings belong to that class. The question then arises of the connection between the embryo and the adult human being, and more specifically about whether the embryo and the adult human being are identical in an ethically relevant way. If we use ‘person’ as a technical term denoting any entity with full moral status, the question can then be rephrased in philosophical terms as a question of whether being a person is a substance sortal or a phase sortal [43]. Phase sortals identify and apply to distinct phases of an object or entity. The (quasi)-biological terms embryo, foetus, infant, child, adolescent, adult are, for instance, phase sortals that apply to human beings as they develop. Substance sortals apply to an object or entity throughout its existence, and if it no longer applies, the object or entity ceases to be that kind of object or entity. Human being is, for instance, a substance sortal, which distinguishes human organisms from dogs, dandelions and dodos.

Personhood theorists hold that ‘person’, in the technical sense we use it here, is either a phase sortal which only applies to human beings when they have developed sufficient cognitive abilities to count as persons, or they hold that person is a substance sortal, but one that only applies to entities fulfilling the criteria for personhood. On the personhood phase sortal account, human beings become persons sometime after birth and may stop being persons sometime before they die, but they only have full moral status in the segment of their lives when they are persons. On the personhood substance sortal account, a new identifiable entity, the person replaces the previous entity, the non-person human infant, when the criteria for personhood are attained.

Those who advocate for the argument from potential reject both of these accounts. It seems rather strange to claim that I only came into existence sometime after my birth and that I am not relevantly identical to myself as a foetus or embryo. Many baby albums now begin with an ultrasound scan of the 12-week-old foetus, and most people would be surprised to hear that that picture and the picture of the newborn are not pictures of the same person as the 18-year-old the pictures are now used to embarrass. Claiming that person in this sense is a substance sortal and that every human life therefore is lived by at least two and, in some cases, three substantively different entities just seems odd. However, claiming a person to be a phase sortal also seems odd because of the link personhood theorists make between this putative phase sortal and full moral status. Conceptualising a person as a phase sortal then has the implication that the moral status of the substantive entity, the human being, changes abruptly in a very significant way. Today, killing me is morally prohibited, but yesterday, when I was not yet a person, it was fully permissible.

The link made between potential and ethical evaluation in the argument from potential agrees that a person is a substance sortal but argues that it is a substance sortal which is co-extensive with the biological substance sortal, human being. From the first moment, a new human organism exists, it is a person. This argument can be made on Aristotelean or Thomistic grounds, e.g., that every human being is a distinct individual with a rational nature, or on grounds of the numerical identity between the embryo and later developmental stages of the same organism. The possibility of monozygotic twinning and chimerism creates problems in relation to identity-based arguments applied to very early embryos, but an exploration of these is outside the scope of this paper [44]. Recently, an argument has been developed by Chunghyoung Lee that “I am not the zygote I came from because a different singleton could have come from it” [45] (p. 295). This argument again creates problems for identity-based arguments in relation to very early embyos, not necessarily for arguments from potential that are not based on strict identity between a zygote or embryo and any specific later human being, but on the premise that a zygote or the embryo has the potential to develop into some human being.

## 4. Applying the Argument from Potential to Stem Cell Models

What are the implications of the argument from potential for the ethical status of hSCBEM? As noted above, we know what the potential of the human embryo is at the type level, because we have irrefutable evidence that human embryos can and often do become born human beings. We also know how human embryos are normally produced, i.e., by the fertilisation of an oocyte by a spermatozoon, and we know a lot about how human embryos develop. The situation is different in relation to hSCBEMs, where we have two important problems in relation to an assessment of the first part of the argument from potential, i.e., whether a specific hSCBEM has the relevant active, intrinsic potential. The argument here will build on previous arguments made by Lewis and Holm in relation to changes in potential caused by the possibility of trophoblast replacement [46], but the argument developed here will rely more explicitly on the account of the argument from potential provided above and identify some more general problems in identifying the potential of a newly developed hSCBEM.

The first set of problems is epistemic. For any given new hSCBEM, we will, at the point in time it is first developed, have much less knowledge and much less certain knowledge about its possible future development than we have for human embryos. The second problem is that whereas the human embryo is a natural, biological organism, the hSCBEM is a techno-scientific artefact and therefore malleable and not naturally circumscribed.

In the literature, it has been suggested by Rivron and co-authors that the first problem can be solved by identifying “tipping points” [47]. They argue that we should use morphological evidence from in vitro development and functional evidence about the possibility to form live and fertile animals from similar animal SCBEMs to generate evidence related to “increased certainty over potentiality”. Here, it might be worth noting in passing that being fertile is not usually taken to be a criterion for moral status, although it may be a criterion for having a perfect SCBEM. The first epistemic problem is related to the morphological evidence. The problem is simply that, at the time, we realise that our newest hSCBEM develops in vitro exactly as a human embryo does, it is too late. If we want to stop the development of new hSCBEMs before they have the potential equivalent to a human embryo, we need to stop the scientific developments when the current hSCBEM does not have that potential. But in order to generate the maximum amount of scientific benefit, for instance in relation to understanding the implantation process, we need to allow developments of new hSCBEMs to get as close as possible to assigning a developmental, morphological identity to the human embryo. At this point in the argument, we could gesture to applying the so-called ‘precautionary principle’, but that principle does not give us any precise guidance on how much precaution to apply. Furthermore, it is not clear that the precautionary principle is a valid principle of rational decision-making, and even if it is a valid principle, it is not clear that it applies to questions of moral status [48,49].

The second epistemic problem is caused by the reliance on evidence from animal SCBEMs for the second tipping point. Here, there is first a problem of specifying what makes an animal SCBEM similar to a human SCBEM, and how to estimate the degree of similarity. Second, there is a much more important problem that identifying a reliable tipping point for potential of hSCBEMs in this way relies on the assumption that the animal model will always be closer to the potential for live birth and later fertility than the similar human model. But there is no obvious reason why this assumption should always be true. If the assumption is not true, the actual tipping point between hSCBEMs that lack potential and those that have it may not be identified until it has already in fact been passed [8,46].

The second main problem in deciding whether a particular hSCBEM has the intrinsic, active potential that is relevant to a determination of moral status is created by the fact that ‘a model’ is a malleable concept. In order to determine the developmental potential of a particular model, we need to define the model precisely and be able to specify which changes to or modifications of the model are compatible with it still being the same model. To use a perhaps trivial example, consider a LEGO model of a particular Ferrari car model. There are many changes we could make to that model that are compatible with it being the same model, e.g., we could add or remove a single brick somewhere or we could exchange all the red bricks with black bricks, but there are also changes we could make that would mean that it was no longer a model of a Ferrari. In relation to a particular hSCBEM, there are modifications we could make that are likely to change the potential of the model, i.e., replacing the hSCBEM trophoblast with the trophoblast of a human embryo [50]. Would that make it into a different model? Both models would be ‘integrated’ as defined by the ISSCR, but they might have considerably different potential. Rivron et al.’s tipping points rely on us being able to specify what demarcates a specific model, since it is only in that case we can use the tipping points to estimate its potential. We could solve this problem by stipulating that any change to an hSCBEM that changes its potential makes it a different model. Such a stipulation would be circular, and it would also require us to be able to specify which changes are potentiality-changing in advance of investigating for tipping points, since the whole purpose of defining the model is to be able to say to a researcher that they now have a new, distinct model for which tipping points need to be investigated. The stipulation would rest on a reasonable ethical distinction, but cannot solve the practical problem of deciding when a model has been changed to such an extent that it is no longer the same model. In relation to implantation research, there is a further problem which is specific to Rivron et al.’s framework since they argue that for our regulatory considerations, we should move from the biological definition of an embryo as “Human cells with the active potential to form a foetus” [47] (Figure 2) to a legal definition which defines the embryo as “a group of human cells supported by elements fulfilling extraembryonic and uterine functions that, combined, have the potential to form a foetus” [47] (p. 3552). This would mean the model we should consider is not only the hSCBEM, but also the model of the uterine lining, and a change in either could change the potential of a ‘Rivron’ model.

## 5. Conclusions

The argument from potential can, if we accept it as valid and sound, provide support for the view that human embryos have very significant moral status from the earliest stages of development and that embryo research should therefore be a topic of regulatory concern. Like most, and probably all strictly philosophical arguments, it cannot explain the exact features of the actual regulation in most jurisdictions. Those features are best explained as the result of a historical, political compromise. Before we formulate new regulations, we may, however, need much more research on the concerns of relevant stakeholders.

If we apply the argument from potential to hSCBEMs, we find that it is in principle applicable. An hSCBEM with the potential to develop into a born human being would have the same moral importance and status as a human embryo, because it has the same potential. But hSCBEMs are techno-scientific artefacts and this means that determining the potential of a newly developed hSCBEM raises a number of epistemic and definitional problems. We have plentiful knowledge of the potential of a human embryo, but very little knowledge about the potential of a new hSCBEM that has just been constructed. We may to some extent be able to predict its potential, but we will not know what the potential is until we have allowed the model to develop. Furthermore, whereas we can precisely identify human embryos, the model concept is malleable and we need a non-arbitrary way of defining which changes to a model also changes its identity from one model to another model. This is not a trivial task. Taken together, these problems undermine regulatory proposals built on distinctions such as the ISSCR distinction between integrated and non-integrated hSCBEM, and Rivron et al.’s identification of morphological and analogical tipping points. They undermine them because there is a realistic possibility that researchers develop an hSCBEM with the same potential as an embryo, before it is realised that that would be an implication of the next step in hSCBEM development; i.e., we cannot rely on the proposed distinctions to identify the developments we need to regulate.

## Data Availability

No new data were created or analyzed in this study.

## Abbreviations

The following abbreviations are used in this manuscript:

hSCBEM	Human Stem Cell-Based Embryo Model
ISSCR	International Society for Stem Cell Research
ESHRE	European Society for Human Reproduction and Embryology
